# Perspective Applications and Associated Challenges of Using Nanocellulose in Treating Bone-Related Diseases

**DOI:** 10.3389/fbioe.2021.616555

**Published:** 2021-05-07

**Authors:** Suliman Khan, Rabeea Siddique, Ding Huanfei, Muhammad Adnan Shereen, Ghulam Nabi, Qian Bai, Sehrish Manan, Mengzhou Xue, Muhammad Wajid Ullah, Hu Bowen

**Affiliations:** ^1^Department of Cerebrovascular Diseases, The Second Affiliated Hospital of Zhengzhou University, Zhengzhou, China; ^2^Department of Hepatobiliary and Pancreatic Surgery, The First Affiliated Hospital of Zhengzhou University, Zhengzhou, China; ^3^State Key Laboratory of Virology, College of Life Sciences, Wuhan University, Wuhan, China; ^4^Key Laboratory of Animal Physiology, Biochemistry and Molecular Biology of Hebei Province, College of Life Sciences, Hebei Normal University, Shijiazhuang, China; ^5^Department of Biomedical Engineering, College of Life Science and Technology, Huazhong University of Science and Technology, Wuhan, China

**Keywords:** nanocellulose, bone diseases, tissue engineering, biodegradation, medical applications

## Abstract

Bone serves to maintain the shape of the human body due to its hard and solid nature. A loss or weakening of bone tissues, such as in case of traumatic injury, diseases (e.g., osteosarcoma), or old age, adversely affects the individuals quality of life. Although bone has the innate ability to remodel and regenerate in case of small damage or a crack, a loss of a large volume of bone in case of a traumatic injury requires the restoration of bone function by adopting different biophysical approaches and chemotherapies as well as a surgical reconstruction. Compared to the biophysical and chemotherapeutic approaches, which may cause complications and bear side effects, the surgical reconstruction involves the implantation of external materials such as ceramics, metals, and different other materials as bone substitutes. Compared to the synthetic substitutes, the use of biomaterials could be an ideal choice for bone regeneration owing to their renewability, non-toxicity, and non-immunogenicity. Among the different types of biomaterials, nanocellulose-based materials are receiving tremendous attention in the medical field during recent years, which are used for scaffolding as well as regeneration. Nanocellulose not only serves as the matrix for the deposition of bioceramics, metallic nanoparticles, polymers, and different other materials to develop bone substitutes but also serves as the drug carrier for treating osteosarcomas. This review describes the natural sources and production of nanocellulose and discusses its important properties to justify its suitability in developing scaffolds for bone and cartilage regeneration and serve as the matrix for reinforcement of different materials and as a drug carrier for treating osteosarcomas. It discusses the potential health risks, immunogenicity, and biodegradation of nanocellulose in the human body.

## Introduction

Nanocellulose refers to the cellulosic materials with dimensions in the nanoscale range, which combines the important properties of cellulose with the features of nanomaterials (Mazhar et al., 2021). It is obtained from plants (de Amorim et al., 2020), produced by different microorganisms predominantly from acetic acid bacteria (Andriani et al., 2020; Khan et al., 2020b), algae (Liu et al., 2017), and animals (Bacakova et al., 2019), as well as synthesized enzymatically such as by the cell-free enzyme systems (Ullah et al., 2015; Kim et al., 2019). There exist different types of nanocellulose, including cellulose nanofibers (CNFs), cellulose nanocrystals (CNCs), and microorganisms-derived cellulose knows as bacterial nanocellulose (BNC) or bacterial cellulose (BC) (Klemm et al., 2018). The CNFs and CNCs are extracted from different plants through complex extraction procedures, involving physico-mechanical degradation and chemical de-structuring such as acid hydrolysis (Dufresne, 2013; Ullah et al., 2016c). BNC is chemically similar to plant-derived cellulose; nevertheless, it represents the purest form of cellulose, as unlike plant cellulose, it does not contain hemicellulose, lignin, and other minerals (Bacakova et al., 2019; Ul-Islam et al., 2019a).

Bacterial nanocellulose is the most prominent biopolymer synthesized by specific bacterial species through the fermentation of sugars and plant carbohydrates. It possesses unique structural, physico-chemical, mechanical, thermal, and biological features and is a Generally Recognized As Safe (GRAS) biological product (Yang et al., 2018; Zhong, 2020). It has been extensively used for various biomedical and other applications in the form of hydrogels, membranes, particles, and three-dimensional (3D) scaffolds, where its properties are tuned according to the target applications (Ul-Islam et al., 2015). Its innate features are modulated through the development of its composites with different materials like natural and synthetic polymers (Shah et al., 2013), nanomaterials (Ul-Islam et al., 2014; Khan et al., 2015; Ullah et al., 2016b), peptides (Curvello et al., 2019), ceramics (DeMello, 2012), clays (Ul-Islam et al., 2013a), plant extracts (Fatima et al., 2021; Ul-Islam et al., 2021a), and others (Mbituyimana et al., 2021). Similarly, its biological synthesis is regulated to produce BNC with controlled features in the form of mechanically and thermally stable hydrogels and form simple to complex 3D structures (Shi et al., 2016). Moreover, its structural features are modified through different pre- and post-synthesis processing approaches (Ul-Islam et al., 2013b). Although the harvesting procedures of BNC hydrogels from the production medium are simple, cost-effective, and time-efficient, the drying procedures such as air-drying, oven-drying, spray-drying, press-drying, critical point drying, and freeze-drying which ultimately determine the characteristic features of BNC, are time-consuming (Lin and Dufresne, 2014; Sharma and Bhardwaj, 2019), and may cause unwanted changes to the innate structure of BNC. For example, air-drying causes the loss of the 3D network structure of BNC due to strong shrinkage-mediated wrinkling, fiber aggregation, and superficial hornification and ultimately results in reduced porosity. On the other hand, critical point drying and freeze-drying, although gentle procedures and retain the original nanofibrous structure (Sharma and Bhardwaj, 2019), may result in complete drying and causes brittleness. BNC is a non-toxic biopolymer that has been found with no severe signs of inflammation and toxicity on the genetic and cellular levels, even in long-term application. Currently, more attention is given to developing BNC-based scaffolds for various medical applications. For instance, the development of BNC-based wound dressings is currently on the market, while some BNC implants are in the phase of clinical studies as a novel solution to yet unresolved complications in regenerative medicine (Klemm et al., 2018; Sharma and Bhardwaj, 2019; Zhong, 2020). Studies have shown that the BNC-based scaffolds play vital roles in the regeneration of bone and cartilage (Khan et al., 2020a), as the drug delivery systems (Pachuau, 2017; Li et al., 2018), biosensing platforms (Jasim et al., 2017; Farooq et al., 2020), diagnostic tools (Ul-Islam et al., 2019b), and several other areas (Islam et al., 2015; Derakhshanfar et al., 2018; McCarthy et al., 2019a, b, Wang et al., 2020a, 2021). The impregnation of nanomaterials to BNC and its use as the wound dressing biomaterial, broad-spectrum antimicrobial, surface disinfectant, and nanodrugs for the treatment of different diseases (Lin and Dufresne, 2014; Di et al., 2017; Wang et al., 2020b) could be an effective approach for addressing several clinical issues.

Bone and cartilage encounter different defects associated with diseases, injuries, and aging. Some of these defects are conventionally treated by using autografts, allografts, and xenografts; however, their general use is hindered by immune rejection. Similarly, osteosarcoma, the malignant bone-tumor accounting for 20% of primary bone cancers (Carina et al., 2019), is another class of bone diseases. The widespread application of currently used strategies for treating osteosarcoma such as surgery (e.g., amputations and bone reconstruction) combined with chemotherapy is limited by the low efficacy of different therapeutic options as well as some chemotherapeutic drugs cause adverse effects and lead to severe complications (Carina et al., 2019). To overcome the adverse effects of surgery and chemotherapy, various biophysical approaches such as sono- and photodynamic therapies, low-intensity pulsed ultrasound, high-intensity focused ultrasound, and hyperthermia have been developed (Sengupta and Balla, 2018; Carina et al., 2019). To date, the knowledge about the efficacy of biophysical therapies against osteosarcoma is limited, and there are only limited reports available about the usage of nanotechnology and nano-delivery systems for the treatment of osteosarcomas (Gu et al., 2013).

The applications of nanotechnology could be expanded toward the treatment of bone and cartilage defects. For example, nanoparticles possess unique properties such as low toxicity, biodegradability, selective tumor cell targeting, higher deposition and retention in tumor tissue, and prolonged blood circulation time (Panday et al., 2018; Zhang et al., 2018b). Although there are also unclear aspects of nanotechnology restricting its broad-spectrum applications, the inherent unique properties of nanomaterials could help in improving their therapeutic efficacy against osteosarcomas (Turnbull et al., 2018; Zhang et al., 2018b). In addition to different nanoparticles, nanocellulose could also be used to replace the affected bone, like in the form of bone substitutes, as a matrix for impregnation of nanomaterials, and as a drug carrier (Khan et al., 2019) for treating different bone-related diseases. For instance, bisphosphonate-modified nanocellulose (pNC) could be used as a bone substitute for bone regeneration. The pNC is a bioresponsive injectable bone material that suppresses the formation of osteoclasts and enhances the differentiation of osteoblasts. Considering the regulation of osteoclast/osteoblast activity, pNC has enormous potential for treating bone diseases (Nishiguchi and Taguchi, 2019). Although the mechanical strength of nanocellulose is quite low compared to that of the bone tissues, it can be improved, such as through crosslinking. In a study, mechanically stable CNCs-based scaffolds were developed through sulfuric acid hydrolysis crosslinking, which showed high compressive strength and porosity. The scaffolds demonstrated *in vivo* osteoinductivity and bone formation across the damaged site (Osorio et al., 2019a). Besides, nanocellulose could also be modified with anti-osteosarcoma metals or nanoparticles, for instance with selenium, strontium, and arsenic nanoparticles, where nanocellulose could not only function as a carrier for different drugs but also serve as a substitute for the damaged bone tissues (Khan et al., 2019; Luz et al., 2020). Both selenium and arsenic have been widely studied for their anticancer effects, where selenium has been found effective against osteosarcoma when doped with hydroxyapatite (Wang et al., 2016; Khan et al., 2019). Therefore, combining these metals with nanocellulose may further increase their utilization in treating bone diseases. Besides bone, nanocellulose could also be used as a substitute for the cartilage that is a smooth elastic tissue and covers the ends of long bones at the joints and nerves and is commonly present in the ear, nose, rib cage, intervertebral disks, and several other parts of the body. Cartilage is softer than bone but much stiffer than muscles and contains chondrocytes which produce a collagenous extracellular matrix. In a study, a highly porous hydrogel comprised of interpenetrating networks of sodium alginate and gelatin reinforced with cellulose nanocrystals was prepared. The composite hydrogel supported the adhesion, growth, penetration, and differentiation of mesenchymal stem cells (MSCs), thus could be used as a substitute for cartilage (Naseri et al., 2016). In another study, a mitogenic hydrogel ink comprised of alginate sulfate with nanocellulose was printed that supported the adhesion, spreading, and proliferation of chondrocyte and produced collagen II (Mller et al., 2017). The printed 3D scaffold could be used as a potential substitute for cartilage.

Literature survey shows that there exist few reviews on using different forms of nanocellulose for their tissue engineering and other biomedical applications. For example, a couple of reviews specifically discusses the tissue engineering applications of BC (Torgbo and Sukyai, 2018) and CNCs (Murizan et al., 2020), while others overview the use of different forms of nanocellulose for bone tissue engineering along with other biomedical applications (Bacakova et al., 2019; Luo et al., 2019; Pang et al., 2020; Subhedar et al., 2021). However, there is no comprehensive review discussing not only the tissue engineering applications but also the role of nanocellulose as a matrix for nanoparticles and drugs for treating osteosarcomas. Moreover, this review discusses the important structural, physiological, and biological features of nanocellulose from the perspective of developing scaffolds suitable for tissue engineering and carriers for drugs and nanoparticles for treating bone defects and cancers. Most importantly, it discusses the immunogenicity and complications associated with the introduction of nanocellulose to the human body.

## Natural Sources of Nanocellulose

### Plants

Plants represent the most abundant source of nanocellulose. It is extracted from different plant sources such as trees, roots, vegetables, grasses, shrubs and herbs, succulents, flowers, and different other plant-derived sources. Trees are the main source of nanocellulose, and it is mostly obtained from coniferous (*Pinus radiata*) (Powell et al., 2016) and leaved trees (birch) (Bacakova et al., 2019). Various other trees, for example, *Khaya senegalensis* (Adewuyi et al., 2018), *Banana pseudostem* (Faradilla et al., 2017), palm and balsa (Bacakova et al., 2019), citrus trees (Matharu et al., 2018), *Syzygium cumini* (Singla et al., 2017), and *Acacia mangium* (Jasmani and Adnan, 2017), are also good sources of nanocellulose. The nanocellulose extracted from coniferous and leaved trees is referred to as the softwood and hardwood-derived nanocellulose, respectively. Hibiscus and cotton are the shrub sources of nanocellulose (Poonguzhali et al., 2017). Various other plant sources of nanocellulose include the corn leaf, carrot, triticale straw, sisal (*Agave sisalana*), *Miscanthus giganteus* (grass), bamboo, pineapple leaf, rice husk, and soybean straw (Song et al., 2018).

### Microorganisms

The microorganism-derived nanocellulose (i.e., BNC or BC) (Yuen et al., 2017) is mainly produced by the Gram-negative acetic acid bacteria belonging to genera *Gluconacetobacter*, *Acetobacter*, *Sarcina*, *Salmonella*, *Achromobacter*, *Agrobacterium*, *Alcaligenes*, *Rhodobacter*, *Azotobacter*, *Pseudomonas*, *Aerobacter*, and *Rhizobium*. Among the different BNC-producing bacterial genera, *Gluconacetobacter xylinum* and *Gluconacetobacter hansenii* are the most widely studied bacterial species (Ahrem et al., 2014; Ullah et al., 2017). Besides, *Gluconacetobacter kombuchae* and *Komagataeibacter medellinensis* (low pH-resistant strain) (Bacakova et al., 2019) are also known for high-quality BNC production. BNC is typically synthesized as pure cellulose by the BNC-producing bacteria, which, unlike the plant-derived cellulose, does not require intensive processing for removal of unwanted impurities or contaminants (Lin et al., 2013). To date, different strategies have been developed for increasing the yield and productivity, minimizing the production cost, and enhancing the structural features of BNC (Ullah et al., 2019c; Ul-Islam et al., 2021b). For example, the addition of yeast extract to the bacterial growth medium enhances bacterial growth, thus leading to high productivity and yield (Saska et al., 2017; Kaminagakura et al., 2019). A study also reported enhanced BNC production through the symbiotic co-cultivation of *M. gisevii* with BNC-producing bacterium (Zharikov et al., 2018). The addition of different supplements to the growth medium not only improves the production but also enhances the structural features of BNC (Ul-Islam et al., 2017, 2020). Different reactors have also been designed for improving yield and productivity (Ullah et al., 2019c). Besides, extensive efforts have been made to explore low-cost substrates and utilize different wastes for cost-effective BNC production (Ul-Islam et al., 2017, 2020).

### Cell-Free Enzyme System

Nanocellulose production by the cell-free enzyme systems represents a relatively new and previously uncharacterized approach. A cell-free enzyme system represents the state-of-the-art conversion of a substrate into the product through a series of enzymatic reactions, each catalyzed by specific enzymes and regulatory proteins (i.e., cofactors) (Khattak et al., 2014; Ullah et al., 2016a). Park and co-workers developed a *G. hansenii*-based cell-free enzyme system through a simple bead beating approach and demonstrated *in vitro* nanocellulose production (Ullah et al., 2015; Kim et al., 2019). The developed system contained all essential enzymes required for the synthesis of cellulose as characterized by the LC-MS/MS-LTQ Orbitrop and SDS-PAGE analyses. The system was further supplemented with external cofactors (i.e., ATP and NAD) to boost the nanocellulose-production (Ullah et al., 2015). The developed cell-free enzyme system effectively produced cellulose at a much higher yield (58%) as compared to the microbial cell system (i.e., 37%) under the same experimental conditions. Moreover, the synthesized cellulose demonstrated superior morphological structure, physiological features, and thermal and mechanical properties, as compared to the cellulose produced by the microbial cells (Ullah et al., 2016c). The cell-free enzyme system represents an *in vitro* and energy-efficient approach for nanocellulose synthesis that can be easily controlled, and the synthesized nanocellulose could be tuned for the desired structural features.

### Algae

*Cladophora* and *Cystoseria myrica* are the major algal species that produce nanocellulose. *Cladophora*-derived nanocellulose is a potential biomedical material due to the presence of several useful materials in its structure, including endotoxins, glucans, and heavy metals (Liu et al., 2017). Its adsorption capacity for Congo-Red-dye (Ruan et al., 2018), hemocompatibility (Rocha et al., 2018), and suitability as the scaffold for cell adhesion and proliferation (Hua et al., 2016) have already been evaluated. Similarly, the *Cystoseria myrica*-derived nanocellulose together with Fe_3_O_4_ has been evaluated for the removal of mercury (Zarei et al., 2018).

### Animals

Nanocellulose has also been obtained from some animals such as *Styela clava* and Halocynthia roretzi Drasche (tunicates from phylum Chordata) (Bacakova et al., 2019). *Styela clava*derived nanocellulose has been used in wound dressings and other biomedical applications, including scaffolds-based tissue engineering and the development of absorbable hemostats and hemodialysis membranes and stitching fibers (Song et al., 2014, 2017). There are also various industrial and technological applications of nanocellulose from animals. For example, Halocynthia roretzi Drasche (composite)-derived nanocellulose together with TiO_2_ nanoparticles was used for the removal of oil from wastewater (Zhan et al., 2018).

## Nanocellulose-Related Health Complications in Humans

Nanocellulose, mainly BNC, is applied in different forms to humans, such as skin substitute (Khan et al., 2018), wound dressing materials (Sajjad et al., 2020), synthetic blood vessels (Scherner et al., 2014), artificial cornea (Wang et al., 2010), bone and cartilage (Basu et al., 2018), synthetic heart valves (Mohammadi, 2011), gastrointestinal tract (Lamboni et al., 2019), tympanic membrane (Kim et al., 2013), dental implants (Jinga et al., 2014), neural implants (Yang et al., 2017), urinary conduit replacement (Huang et al., 2015), contact lenses (Cavicchioli et al., 2015), and several others, and therefore, it is necessary to identify the associated potential health risks (Endes et al., 2016). Several studies have reported brown lung, alveo-bronchiolitis, fibrosis, and granulomatous inflammation *in vivo* following the exposure to cellulose (Ttrai et al., 1995). Similarly, studies have reported the persistence of cellulose, both *in vitro* and *in vivo*, alveolitis, granulomata, and higher cellulose fiber durability in the lungs (Endes et al., 2016). A study has also reported the cytotoxicity of high doses of nanocellulose crystals (Yanamala et al., 2014). The high doses of cellulose can significantly increase the radical formation (Stefaniak et al., 2014) and generation of oxidative stress during long-term exposure, which can ultimately lead to serious consequences in the human body. Currently, few studies have investigated the genotoxic effects of nanocellulose in the human body, including potential for mutagenicity, formation of micronuclei, and measurement of DNA strand breaks (Endes et al., 2016). Although no studies have reported any effects on the formation of micronuclei or alteration in DNA quality after exposure to nanocellulose exposure (Kovacs et al., 2010; Cataln et al., 2015), a study by de Lima et al. (2012) found chromosomal aberration in the animal cells after exposure to nanocellulose. Surprisingly, the chronic exposure to nanocellulose *via* inhalation increased the susceptibility to cytotoxicity in males and was associated with higher inflammatory and oxidative stress. These biochemical alterations further cause significant genotoxicity (Shvedova et al., 2016), and the genotoxic effects are extremely detrimental to the male reproductive system (Farcas et al., 2016). However, a detailed long-term mechanistic toxicological assessment study to investigate the potential biological effects on human health is still lacking. These assessments are essential and are considered as the landmarks of nanotoxicological research strategies (Camarero-Espinosa et al., 2016).

## Attractions in Nanocellulose-Based Scaffolds to Substitute Bone Tissues

As bone primarily maintains the shape of the body, the biological scaffolds serving as the bone substitute must demonstrate the desired mechanical strength and biocompatibility. Nanocellulose possesses unique structural, physico-chemical, mechanical, thermal, and biological features to meet the desired features of biological scaffolds. In addition, its 3D fibrous and porous structure and the presence of free hydroxyl (OH) groups on its surface provide an excellent platform for the development of biomaterials with tuned properties.

### Abundance and Renewability

Nanocellulose is the most abundant and renewable material on Earth. As described earlier (see section Natural Sources of Nanocellulose), it is obtained from a variety of sources like plants, microorganisms, algae, and animals and synthesized enzymatically. The abundance, renewability, and easy availability, along with other properties, make nanocellulose an ideal choice for the development of biological scaffolds for applications in bone tissue engineering (Table 1).

**TABLE 1:** Applications of BNC-based biomaterials in bone regeneration.

Reinforcement material	Synthesis method	Model system for analysis	Enhanced properties	References
HAp	Post-synthesis phosphorylation	Ca/P ratio	High Ca/P ratio	DeMello, 2012
	Loading of HAp to BNC	HAp loading	High loading of HAp in phosphorylated BNC	Wan et al., 2007
	Post-synthesis loading	Ca/P ratio, *in vivo* inflammatory tests	Ca/P ratio similar to natural bone and no *in vitro* inflammation	Saska et al., 2011
	Biomimetic synthesis	hBMSC	Enhanced cell adhesion and biological activity	Fang et al., 2009
	Post-synthesis loading	XPS analysis, ALP activity, osteoblast growth, and formation of bone nodule	Presence of Ca^2+^ and PO_4_^2^, enhanced adhesion growth of osteoblast, and osteoconductivity on membranes	Tazi et al., 2012
HAp and magnetic nanoparticles		Ca/P ratio, crystallinity, magnetic field response, *in vitro* MC3T3-E1 cells	High porosity, decreased crystallinity and swelling, decreased saturation magnetization, and enhanced biocompatibility	Torgbo and Sukyai, 2019
HAp and graphene oxide	Wet chemical precipitation	ALP activity, growth of MG-63 and NIH-3T3 cells	Water uptake, *in vitro* degradation, cell adhesion and growth, and ALP activity	Ramani and Sastry, 2014
HAp and gelatin	Laser patterning	Porosity and *In vitro* C5.18 cells	Enhanced adhesion and proliferation of chondrogenic rat cells, high porosity	Jing et al., 2013
HAp and strontium	Oxidation of BNC, *ex situ* mineralization	*In vitro* cytotoxicity and hemocompatibility	Guided bone regeneration, *in vivo* biocompatibility, *in vitro* degradation, bioactivity, non-cytotoxicity, low inflammation, swelling, thermal stability, enhanced desorption	Luz et al., 2020
HAp or Col with and without OGP	Post-synthesis loading	CHO-K1 cells, CBMN assay, comet assay, XTT assay, and clonogenic assay	Cell adhesion and proliferation, and no mutagenic, genotoxic, or cytotoxic effects on the cells	Raquel Mantuaneli Scarel-Caminaga, 2014
HAp and poly (vinyl pyrrolidone)	Biomimetic mineralization	Ca/P ratio	Enhanced mineralization	Yin et al., 2011
Agarose, gelatin, HAp, and procyanidins	Post synthesis crosslinking	Mechanical strength, pore size distribution, *in vitro* hGMSCs, *in vitro* and *in vivo* bone formation	Porosity, mechanical strength, cell viability, *in vitro* bone formation in mice and *in vivo* bone repair in rabbit	Huang et al., 2017
GO, Hap, and -glucan	Free radical polymerization and freeze-drying	Surface morphology, porosity, and mechanical strength, hydrophobicity, aqueous degradation, *in vitro* MC3T3-E1	High stability, hydrophobicity, aqueous degradation, spongy morphology, porosity, and mechanical strength, antibacterial activity, biocompatibility, hemocompatibility	Umar Aslam Khan et al., 2020
2-chloro-*N, N*-dimethyl ethylamine hydrochloride, glycidyl trimethyl ammonium chloride, and monochloro acetic acid sodium salt	Post-synthesis chemical reaction	*In vitro* EqMSCs	Enhanced *in vitro* adhesion, proliferation, and osteogenic differentiation of EqMSCs	Favi, 2014
Gelatin	Post-synthesis loading	Crystallinity index, mechanical strength, *in vitro* adhesion of NIH 3T3 cells	Crystallinity index, enhanced mechanical strength and thermal stability, improved *in vitro* cell adhesion	Cai and Kim, 2010
PVA and boron nitride	3D printing	Mechanical strength, swelling, *in vitro* osteoblast cell line	Decreased tensile strength and increased elongation strain, enhanced cell adhesion and viability, improved swelling	Aki et al., 2020
Plant-derived recombinant human osteopontin (p-rhOPN), and RGD-containing biomolecule	*In vitro* grafting	Quantification of p-rhOPN immobilization, *in vitro* mineralization, and *in vitro* hPDLSCs	Enhanced osteogenic differentiation of hPDLSCs, cytocompatibility, *in vitro* calcification	Klinthoopthamrong et al., 2020
Bone morphogenic protein (BMP-2)	Post-synthesis loading	*In vitro* mouse fibroblast-like C2C12 cells, *in vivo* bone formation	Differentiation of C2C12 cells into osteoblasts and *in vivo* formation of bone with high calcium content	Shi et al., 2012a, b
	3D scaffolds, ECM-mimicking	Low dose treatment of BMB-2, micro- and nano-porosity, *in vitro* C3H10T1/2 cells	Enhanced cell adhesion, growth, and infiltration, bone matrix secretion and maturation, biomineralization, osteoinduction	Dubey et al., 2020
Collage and BMP-2	Malaprade and Schiff-base reactions, template method combined with reverse-phase suspension regeneration	Porosity, *in vitro* MC3T3-E1, ALP activity	Biocompatibility, 3D porous microspheres with multiple structures, thermal stability, increased crystallinity, osteoblast differentiation	Zhang et al., 2020
Otoliths and collagen	Post-synthesis loading	Histological examination	In vivo regeneration of bone tissue with higher osteoblast activity, degree of regularity, and osteo-reabsorption activity	Olyveira et al., 2011
Col1	Post-synthesis crosslinking	Tensile strength, elastic modulus, and morphology and proliferation of osteogenic cells	Decreased tensile strength and elastic modulus of BNC-Col1, a slight increase in strain at break, cell viability and proliferation, and maintenance of cell morphology on the scaffold	Saska et al., 2012
Paraffin wax particles	*In situ* loading of particles	MC3T3-E1 osteoprogenitor cells, confocal microscopy, and histology	Enhanced clustering of MC3T3-E1 osteoprogenitor cells in the porous composite	Zaborowska et al., 2010

*An overview of different reinforcement materials, synthesis methods, model systems used for analysis, and enhanced properties of BNC and its composites.*

### Surface Chemistry

Nanocellulose has a high surface area and aspect ratio (length to diameter) and contains abundant OH groups. The cellulose chain is asymmetric, with one end having reducing functionality due to the presence of the OH group while the other end is non-reducing (Habibi et al., 2010; Islam et al., 2018). On average, each glucose monomer in cellulose contains three OH groups, which can be easily accessed for surface modification (Phanthong et al., 2018). For example, TEMPO-oxidation at the surface of nanocellulose creates a negative charge by replacing the OH groups with carboxylic (COOH) and aldehyde (CHO) groups, which allows the repulsion of nanofibrils and causes fibrillation (Saito et al., 2007; Isogai et al., 2011), thus greatly contributes to addressing the aggregation issue of cellulose fibrils. This aggregation issue could also be addressed by introducing the anionic sulfate groups onto the surface of nanocellulose that produces electrostatic repulsion among the fibers in the aqueous dispersion and yield stable CNCs (Eyley and Thielemans, 2014). It is worth mentioning here that not all OH groups are modified; a large number of reactive OH groups are preserved to allow the grafting or doping of other molecules to impart additional structural and functional characteristics to nanocellulose. A study reported the uptake of non-aggregated softwood pulp sheet-derived nanocellulose by different cells (Hosseinidoust et al., 2015), thus could be used as a carrier for nanodrugs.

### Biocompatibility

Biocompatibility refers to the ability of a material to interact with living tissues without provoking the immunogenic response, inflammation, or allergy and remain non-toxic (Morais et al., 2010; Ul-Islam et al., 2015). According to Williamss definition, biocompatibility is the ability of a material to perform with an appropriate host response in a specific application (Williams, 2019). In general, all types of nanocelluloses are considered biocompatible materials in that these are non-toxic, non-immunogenic, non-inflammatory, non-allergic, and somehow supports the adhesion, proliferation, growth, migration, and differentiation of cells, either alone or in the form of composites with other materials (Eslahi et al., 2020). A study reported the use of bleached birch pulp-derived nanofibrillated cellulose for wound dressing, which effectively adhered and detached from the skin after completion of wound healing (Hakkarainen et al., 2016). Similarly, BNC is considered a moderate to highly biocompatible biomaterial due to its non-toxic nature and ability to support cell growth, proliferation, and infiltration owing to its highly porous and fibrous structure (Klemm et al., 2001; Schumann et al., 2009). An *in vivo* study demonstrated the subcutaneous implantation of BNC in rats up to 12 weeks that did not show any signs of immunogenicity, inflammation, or formation of exudates around the implant (Helenius et al., 2006). Although non-toxic, pristine BNC lacks cell adhesion sites and its biocompatibility is not up to the desired levels in some cases; thus, different strategies like a surface modification to introduce bioactive functional groups such as peptides antimicrobial peptides (Frsatz et al., 2018) and formation of composites with compatible polymers like gelatin (Khan et al., 2018), chitosan (Ul-Islam et al., 2019b), collagen (Zhang et al., 2020; Li et al., 2021), and other materials, have been developed to achieve the desired biocompatibility for specific applications (Phanthong et al., 2018; Ullah et al., 2019b; Table 1).

### Biodegradability/Bioresorbability

Biodegradation or bioresorbability is an important feature of nanocellulose for its application in bone tissue engineering (Ullah et al., 2019b). The nanocellulose-based scaffolds degrade both *in vitro* and *in vivo*; nevertheless, these sometimes require additional pre-treatment and modification, such as oxidation (Faradilla et al., 2017), and must degrade at a controlled rate of resorption for specific bone tissue engineering applications. Moreover, the degradation products of nanocellulose are also biologically safe due to their innate non-toxic nature; however, a careful selection of the reinforcement materials during the development of nanocellulose-based scaffolds must be ensured for the safety of the degradation products. A study reported that the architecture of biological scaffolds changes with the degradation, where the produced byproducts could interfere with other *in vivo* biological processes (Dorozhkin, 2013).

Compared to other forms of nanocellulose, BNC is rarely biodegradable due to its crystalline nature and compact fibrils arrangement. Moreover, human lacks the cellulose-degrading cellulase enzyme. The biodegradation of BNC has been enhanced through several methods. For example, Li et al. (2009) carried out the periodate oxidation of BNC, which led to its enhanced *in vitro* oxidation in water, phosphate buffer saline, and simulated body fluid. The periodate oxidation of BNC allows specific cleavage of C2C3 of the glucopyranoside and produces two aldehyde molecules from a single glucose unit as well as the biodegradable and biocompatible 2,3-dialdehyde cellulose (DAC) (Fan et al., 2001; Li et al., 2009). The DAC is biodegradable at physiological pH, both *in vitro* and *in vivo*, and produces 2,4-dihydroxybutyric acid and glycolic acid (Singh et al., 1982), where the former participates in the metabolism of L-homoserine in the liver while the latter is either excreted through urine or enters the Krebss cycle (RoyChowdhury and Kumar, 2006). Similarly, a study by Yang et al. (2016) reported the development of carboxymethylated chitosan and hairy nanocrystalline cellulose-based biodegradable aerogels.

### Immunogenicity

Immunogenicity refers to the potential of a substance, referred to as antigen, to provoke an immune response when it enters the body. Although nanocellulose is generally perceived as non-immunogenic, very little knowledge about the immunogenic impact of nanocellulose on the immune cells such as macrophages and dendritic cells is available. The evaluation of immunogenicity is particularly important when nanocellulose-based scaffolds are used for *in vivo* tissue engineering applications or when these come in direct contact with the blood. A very recent review provides the current-state of knowledge of the immunological aspects of nanocellulose against the immune cells (oli et al., 2020). A study reported that CNCs induce inflammation upon internalization by macrophages; nevertheless, this immunogenic response by the macrophages can be suppressed by introducing special functional groups on nanocellulose. For example, the introduction of carboxyl groups onto the surface of CNFs shifted the tolerogenic potential of dendritic cells toward the induction of regulatory CD8^+^ T cells. On the other hand, the introduction of phosphonates onto the surface of CNFs enabled the dendritic cells to induce both the regulatory CD8^+^ T cells and type I regulatory cells (Tomi et al., 2018). These and several other studies show that nanocellulose, especially CNCs, can potentially induce an immunogenic response; nevertheless, this response and its level are highly dependent on the source, preparation methods, morphology and size, agglomeration, presence of contaminants in nanocellulose as well as the type of interacting cells.

### Hemocompatibility

Blood compatibility, known as hemocompatibility, refers to the ability of a biomaterial to interact with the blood without causing toxic effects. Hemocompatibility is an important property of biomaterials for the development of blood-contacting artificial organs, such as artificial heart valves (Mohammadi, 2011) and blood vessels (Scherner et al., 2014). The scaffold, which needs to be implanted into the human body, must be hemocompatible as non-hemocompatible scaffolds may cause toxic effects at the site of implantation such as inflammation, provoking an immune response, or infection. The nanocellulose-based scaffolds are generally hemocompatible (Andrade et al., 2011; Leito et al., 2013) and allow proper osteoconduction, integration, and induction (Pang et al., 2020). The hemocompatible nature of BNC was first reported by Andrade et al. (2011), who determined the plasma recalcification time and whole blood clotting of RGD-modified BNC. The findings of this study showed the successful deposition of plasma protein and prevention of platelet adhesion both on pristine and RGD-modified BNC scaffolds, indicting the hemocompatible nature of BNC (Andrade et al., 2011). In another study, Leito et al. (2013) evaluated hemocompatibility of BNC and polyvinyl alcohol (PVA) nanocomposite by determining the whole blood clotting time, plasma recalcification, Factor XII activation, platelet adhesion and activation, and hemolytic index. The findings of the study showed low activation of platelets and Factor XII, indicating the hemocompatible nature of BNC/PVA composite (Leito et al., 2013). In a more recent study, Osorio et al. (2019b) compared the *in vitro* and *in vivo* hemocompatibility of 3D BNC scaffold with the 2D BNC architecture. The findings of the study showed antihemolytic and anti-thrombogenic effects and only a mild acute inflammatory response of BNC (Osorio et al., 2019b). The hemocompatible BNC-based scaffolds promote neo-vascularization, gaseous exchange, and diffusion of nutrients and minerals in the newly formed or regenerated bones (Seyednejad et al., 2012; Torgbo and Sukyai, 2018). A study showed that the oral administration of TEMPO-oxidized cellulose nanofibers in mice effectively reduced the concentrations of postprandial glucose-dependent insulinotropic-polypeptide, plasma insulin, triglycerides, and blood glucose, suggesting the occurrence of cellulose-induced significant biological and hemocompatible activities (Lin and Dufresne, 2014).

### Mechanical Strength

The cellulose fibers containing strong hydrogen bonding networks and various OH groups offer unique surface properties (Dufresne, 2017; Klemm et al., 2018). The cellulose fibrils contain the crystalline (highly ordered regions) and amorphous (disordered regions) structures (Cheng et al., 2009). The chain molecules at the crystalline regions are packed in such ways that these significantly enhance the stiffness and strength of cellulose, while the amorphous regions confer flexibility (Klemm et al., 2018). Besides, the low density (1.6 g/cm^3^), lightweight, and other strength properties make nanofibers highly stiff of 220 GPa of elastic modulus (greater than Kevlar fiber) and high tensile strength of 10 GPa (greater than cast iron), and the strength ratio to weight is eight times greater than the stainless steel. The mechanical strength of nanocellulose is further enhanced by developing its composites with mechanically strong reinforcement materials such as ceramics, nanoparticles, and polymers. The nanocellulose-based composites possess excellent mechanical properties due to their transparent nature and lightweight (Abdul Khalil et al., 2012; Abitbol et al., 2016). For example, the soybean-derived nanocellulose impregnated with three different types of synthetic polymers showed excellent mechanical properties, including stiffness and tensile strength as compared to the pristine nanocellulose (Wang and Sain, 2007). In another study, Nogi et al. (2009) studied the fabrication of wood flour-derived nanocellulose-based transparent paper and found a high modulus (13 GPa) and strength (223 MPa) and minimal thermal expansion (8.5 ppm K^1^) of the optically transparent nanocellulose paper.

### Porosity

Porosity is one of the most important properties of biomaterials, specifically for their applications in bone tissue engineering. A porous material promotes the effective release of biofactors such as cells, drugs, nanomaterials, proteins, and others from the scaffold. From the biomedical perspective, porosity is one of the most important features of nanocellulose as it allows the impregnation of different minerals, particles, ceramics, polymer solutions, and viable cells (Li et al., 2021).

Bacterial nanocellulose is porous in nature, and its level of porosity is largely dependent on its synthesis method, chemical composition of the medium, microbial strain, pre- and post-synthesis processing, and drying methods. The porous geometry of BNC provides an ideal environment for the impregnation of a variety of materials into its matrix, including solid particles of different shapes as well as liquid solutions (Shah et al., 2013). The innate porous nature of BNC could be advantageous from the perspective that it would prevent the invasion of microbial cells; however, the small pore size could be a limitation in that it may prevent the impregnation of large size particles and infiltration and migration of mammalian cells (Hutchens et al., 2006). The porous morphology of nanocellulose is also advantageous for the targeted drug delivery, as well as it serves as an efficient physical barrier against external infections (Kowalska-Ludwicka et al., 2013). Although the innate properties of BNC favor its application as a scaffold for bone, cartilage, and connective tissue formation, its small pore size prevents the infiltration of cells deep into its matrix, thus limiting its direct application. The pore size of BNC could be controlled at micro, nano, and mesoscales to meet the desired features for bone tissue engineering application. One such strategy is the introduction of various materials, called porogens (Khan et al., 2016). Examples of porogens include different salts, paraffin particles, ice crystals, gelatin, and different sugars, which are added to the network structure of BNC as the space holders and subsequently removed, leaving behind pores of desired shape and size (Bckdahl et al., 2008). The porogens should be adsorbed only physically and not chemically, whose removal should not alter the fibrous morphology of BNC. The porogens of the desired shape and size are selected according to the size of the cells and the end application of nanocellulose. In one study, Bckdahl et al. (2008) used the potato starch and paraffin wax particles as the porogens for the development of interconnected BNC tubes. They achieved partial particle fusion through heat treatment of paraffin wax particles at specific temperatures. The developed BNC tubes supported the growth of smooth muscle cells inside the pores (Bckdahl et al., 2008). In another study, gelatin was added as porogen to BNC to regenerate 3D microporous regenerated scaffolds. The developed scaffolds supported not only the adhesion and proliferation of cells but also their impregnation deep into the matrix of the scaffold, thus suggesting the development of 3D scaffolds. The developed porous scaffolds also showed considerable *in vivo* results in the mice model (Khan et al., 2018). In a study, a highly porous nanocomposite of BNC was developed through the incorporation of Fe_3_O_4_ and hydroxyapatite (BNC/Fe_3_O_4_/HAp) (Torgbo and Sukyai, 2019). The porosity of the nanocomposite was comparable to that of the trabecular/cancellous bone. Moreover, the BNC/Fe_3_O_4_/HAp nanocomposite demonstrated high mechanical strength and cytocompatibility, thus demonstrating its ability to promote bone tissue regeneration.

### Electric Charge and Wettability

The electric charge and wettability, as well as the charge density of nanocellulose, play an important role in the biotechnological properties of biomaterials for their applications in tissue engineering. Nanocellulose can be desirably modulated by adding different chemical groups, and its structural properties could be tuned to meet the desired features of bone tissues. Generally, moderate wettability modulates the cell growth and adhesion or adsorption (Bacakova et al., 2011), while the charge density modulates the roughness and morphology of nanocellulose films (Ahola et al., 2008) and their interaction with the cells to improve the cell adhesion, growth, and susceptibility for transfection with DNA constructs (Liu et al., 2016).

## Applications

### Nanocellulose as a Substitute for Bone and Cartilage Regeneration

The repairing of cartilage damage resulting from trauma or degeneration has been a serious clinical concern (Das et al., 2019). The available treatments for small cartilage defect repair include multiple drilling, abrasion arthroplasty, mosaicplasty, and autogenous and allogeneic chondrocyte transplantation (Cao et al., 2014). There are several limitations associated with the use of allografts, such as disease transmission, immune rejection, and slower remodeling. Likewise, autografts have limitations for their requirements of the patient to undergo many surgeries (Baldwin et al., 2019). The rise of tissue engineering that utilizes cells, biodegradable scaffolds, and growth factors provides a new avenue for the repair of articular cartilage (De Witte et al., 2018). In cartilage tissue engineering, scaffolds provide a 3D structure for cartilage cells and support cell adhesion and proliferation (De Witte et al., 2018; Kabir et al., 2020). The scaffolds are further supplemented with different growth factors or antimicrobial agents, which help in encountering microbial and other infections (Ul-Islam et al., 2014). The structure of a cartilage scaffold is required to mimic the native articular cartilage, which is an oriented structure associated with its mechanical function. The oriented ECM-derived scaffolds enhance the biomechanical property of tissue-engineered cartilage, while the oriented poly(lactide-co-glycolide) (PLGA) scaffolds efficiently promote cell migration, thus probably contributes to improving tissue regeneration. The physical and biochemical properties are crucial for the scaffolds during the entire cartilage repair process (Dorati et al., 2017; Kabir et al., 2020).

**FIGURE 1:**
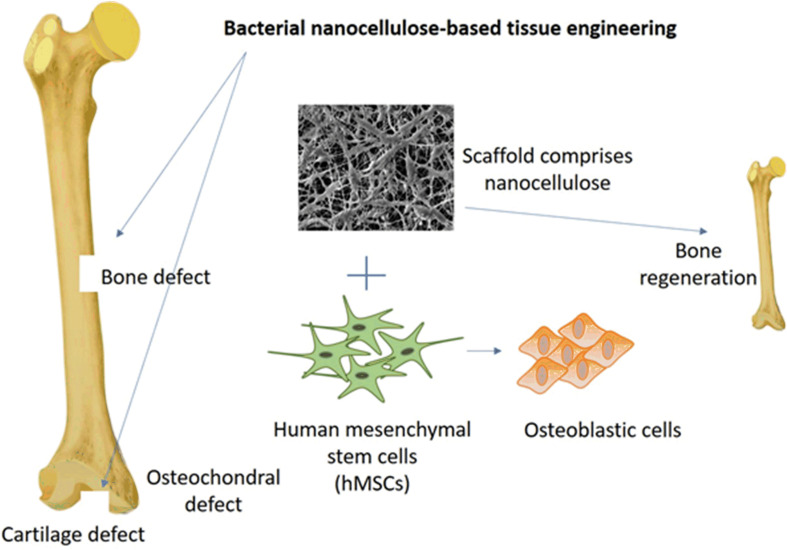
Bone regeneration concept conducted by the synergistic effect of hMSCs cells and BNC-based scaffold. The scaffold allowed the differentiation of hMSCs into the osteoblasts.

The scaffolds comprised of nanocellulose have a great efficacy for bone and cartilage regeneration and allows various types of cells to adhere and develop into the desired tissues and organs (Figure 1; Halib et al., 2017). A study reported that a membrane comprised of BNC and HAp was used to regenerate bone by significantly enhancing the adhesion and proliferation of osteoblastic cells with elevated bone nodules due to higher activity of phosphatase (Tazi et al., 2012). Studies have also reported the development of BNC and collagen composite. The developed scaffold supported the *in vitro* adhesion, growth, and migration of cells. Further, the developed scaffolds showed the regeneration of pig meniscus. The *in vitro* and *in vivo* studies show the potential use of BNC/collagen scaffolds in meniscus transplantation (Bodin et al., 2007) and artificial cartilage (Lopes et al., 2011) for treating articular joints. In a recent study, BNC was used to design an ear-shaped structure that could be utilized to develop a complete human ear with a specific size and shape (Nimeskern et al., 2013). *In vivo* evaluation of non-critical bone defects in rat tibiae on BNC membrane showed no inflammatory reactions with defects completely filled by the new bone tissues after 4 weeks (Saska et al., 2011). Similar results were obtained from studies on the bone regeneration effect of BNC membrane on rat skulls, which showed new bone formation on the margin and center of the bone defect after 8 weeks of the implant (Lee et al., 2017b). Another study conducted using healthy male beagle dogs showed healing of implant sites with no evidence of inflammatory reactions and implant failure (Lee et al., 2017a). The evaluation of BNC as a barrier membrane for guided bone regeneration on rat calvarial defect did not induce any inflammation and maintained adequate space for bone regeneration (Lee et al., 2015). Similarly, no foreign body reaction was observed when BNC was grafted to correct the nasal dorsum of rabbits, which showed a positive sign of BNC integration by fragmentation after 6 months (Amorim et al., 2009). Mineralized bone formation was observed both on the outer and inner surface of femoral cortical bone in dogs on day 14 of the implant (Yuan et al., 2006). In a study, BNC was used as a guided tissue regeneration membrane to repair maxillary canine periodontal defects in beagle dogs, which showed enhanced periodontal tissue regeneration and formation of new bone (Zhang et al., 2018a). It could be concluded from the above studies that the different nanocellulose-based scaffolds could be efficiently used in *in vivo* regeneration of different types of bones, including tibiae, skulls bones, calvarial defected bone, nasal dorsum bone, femoral cortical bone, tendon, and periodontal tissue regeneration through the formation of new bone (Jiang et al., 2020; Zhu et al., 2020). Typical bone regeneration by using BNC-based scaffold as the repair material is illustrated in Figure 1.

### Nanocellulose as a Matrix for Doping of Minerals and Ceramics

In order to enhance the potency, accessibility, and stability of nanomaterials, such as HAp, silica, calcium carbonate (CaCO_3_), calcium chloride (CaCl_2_), and nanoclay, for their application in bone tissue engineering, these are impregnated into the fibrous network or blended with different types of nanocellulose whether these interact chemically with the free OH group or adsorb physically between the fibers. For this purpose, various strategies have been developed, such as *in situ* addition of nanomaterials to the culture medium (Shah et al., 2013), *ex situ* vigorous stirring of nanocellulose with the nanoparticles suspension (Zhang et al., 2015), and solvo (hydrothermal) approach where cation adsorption of cellulose is carried out (Shahmohammadi Jebel and Almasi, 2016). The addition of CaCO_3_ to the bacterial growth culture helps the binding of nano metallic oxides to bind with BNC (Mohammadkazemi et al., 2016). Similarly, the soaking of BNC in an ethanol solution of silicon ethoxide enhanced the uptake of silica nanoparticles and the development of BNC-SiO_2_ nanocomposite (Barud et al., 2008; Sai et al., 2013). To produce iron oxide containing BNC nanocomposite, the solvothermal technique was applied by autoclaving urea, BNC, and iron nitrate (Wan et al., 2015a, b). The solvothermal technique using ethanol or hot water was used to develop 30 nm sized CdS/BNC nanomaterials (Yang et al., 2012). To develop pure metallic nanoparticles and BNC/metal nanocomposites, two different approaches are used: either readymade metallic nanoparticles are mixed with BNC, or BNC itself is used as the reducing and capping agent for the generation of metallic nanoparticles from metallic salts. This approach has been effectively used for the production of Pd, Au, and Ag nanoparticles (Li et al., 2008; Johnson et al., 2011). BNC can easily cause a reduction of silver or gold salt solutions at 55100C and produce BNC-capped silver and gold nanoparticles with enhanced availability and stability when exploited for variable applications. Although the *ex situ* impregnation of already prepared metallic nanoparticles into the BNC matrix can be achieved through simple stirring, the *in situ* uptake by the microbial cells requires chemical or physical treatment such as ultrasonic activation (Cai et al., 2011).

Hydroxyapatite, obtained from various sources, including eggshells and bones of animals or codfish, is considered as the efficient carrier for the nanodrugs due to its biocompatibility and hydrophilic nature and has been extensively used in bone tissue regeneration applications (Wang et al., 2011; Luo et al., 2014; Ramani and Sastry, 2014; Kong et al., 2016; Ullah et al., 2018, 2019a, 2020; Table 1). An earlier study reported the formation of calcium phosphate in the BNC matrix, which supported the adhesion and proliferation of osteoblast cells as well as the differentiation of mesenchymal stem cells into bone cells in the absence of external markers (Fang et al., 2009). A study by Zimmermann et al. (2011) reported the development of calcium-deficient HAp composite, which supported the adhesion of osteoprogenitor cells. Although the surface bioactivity of HAp causes the lack of biofunctionality of the implanted drug (Meagher et al., 2016), several studies have concluded that HAp takes the drug into the bloodstream instead of delivering it to the target site (Kundu et al., 2013; Sun et al., 2018). The drug released in the bloodstream can cause adverse effects and activate the immune cells, which eliminate the drug from the body prior to reaching the target site. Other limitations include low stability, high pH sensitivity, diverse chemical composition, and ionic surface, which may cause alteration in the drug composition or poor delivery of a drug (Huang et al., 2017; Veerla et al., 2019).

The mineralized tissues also contain CaCO_3_ and CaCl_2_. Earlier studies reported the deposition of CaCO_3_ into the BNC matrix by using sodium carbonate and CaCl_2_ as the reagents and in the presence of microwave irradiation (Stoica-Guzun et al., 2012, 2013). In another study, Saska et al. (2011) developed a BNC composite with CaCl_2_ and sodium hydrogen phosphate that effectively regenerated a defect in rat tibial bone by completely regenerating the bone within 4 weeks of implantation and did not show any inflammation.

### Nanocellulose as a Drug Carrier for Treating Bone-Related Diseases

Various novel approaches have been developed to treat bone-related diseases, among which the use of nanodrugs is receiving great attention due to their broad-spectrum antimicrobial and antitumor activities. For example, the CaCO_3_, copper, silica, gold, magnesium-oxide, silver, and boron nanoparticles have been reported to effectively treat various bone-related diseases, such as multiple myeloma, rheumatoid arthritis, microbial infections, and osteoporosis (Bari et al., 2017; Hassani Besheli et al., 2017; Qadri et al., 2017; Gisbert-Garzarn et al., 2020; Cmara-Torres et al., 2021). In a study, the administration of gold nanoparticles in the ankle of rats effectively reduced collagen-induced arthritis and inhibited angiogenesis by blocking the key factors, such as vascular endothelial growth factor (VEGF), synovial fluid, and cell proliferation (Tsai et al., 2007). Besides the broad-spectrum therapeutic ability, nanodrugs have some limitations. For example, metallic nanoparticles might activate the immune system and immune responses and lead to the removal of nanodrug by the mononuclear phagocytic system, Kupffer cells, and phagocytic macrophages (Dong et al., 2012). The macrophage and phagocytosis removal of metallic nanoparticles could be avoided if their size is equal to or more than 100 nm and these are hydrophilic in character (Dong et al., 2012).

After injecting in humans, the nanodrugs are often taken up either by the Kupffer cells or hepatocytes. In Kupffer cells, the nanodrugs are degraded, while hepatocytes eliminate them through the hepatic biliary duct. For example, the PLGA nanoparticles can deliver the drugs; however, their retention time is very low in the bloodstream, and the drug is either eliminated by the Kupffer cells or hepatocytes due to their hydrophobic nature (Adjei et al., 2016). Even if the PLGA nanoparticles escape the Kupffer and hepatic cells and are delivered to the target site, these cause acidification in cells after degradation and give rise to the generation of reactive oxygen species (ROS) (Dos Reis et al., 2019). In a study, Yan et al. (2013) investigated the toxic effect of PLGA nanoparticles on the retinal pigment epithelium (RPE) cells and indicated that PLGA nanoparticles caused 20% apoptosis of RPE cells, and the apoptosis frequency reached 50% at higher concentration.

The free OH groups in BNC possess strong hydrogen bonding due to their hydrophilic nature; therefore, these serve as the ideal candidate for interaction with drugs and their controlled delivery (Ptzinger et al., 2017). The encapsulation of nanodrug in BNC can increase the size of nanoparticles and, due to the hydrophilic nature of cellulose, can skip the immune response. Various cell lines, such as HBMEC, bEnd, RAW 264, MCF-10A, MDA-MB-231, MDA-MB-468, KB, PC-3, and C6 were assessed for the cytotoxic effect of nanocellulose, where none of the cell lines were affected after 48 h (Pachuau, 2017). The fluorescein-5-isothiocyanate technique was used to label the nanocellulose to elucidate the site-specific uptake that resulted in minimal imprecise cellular receive, indicating nanocellulose a useful candidate for drug delivery (Dong et al., 2012; Pachuau, 2017). The coating of BNC on tablets using the spray technique enhanced the mechanical properties of tablets with the prolonged drug release (Amin et al., 2012). In a similar study, berberine hydrochloride and berberine sulfate coated with BNC resulted in drug protection with increased drug release duration (Huang et al., 2013). The boding of cell-specific antigen and receptor with nanocellulose and nanodrugs can further enhance the cell-specific uptake ability of the drug (Tan et al., 2019). In a recent study, Li et al. (2018) reported the formation of a sandwiched structure of BNC with polyaniline that showed sustained drug release at varying pH and under electrical stimulation (Li et al., 2018). These studies demonstrate the potential use of different forms of nanocellulose and their composites as drug delivery systems for treating bone-related diseases.

## Challenges

### Challenges Associated With Nanocellulose Production and Purification

Nanocellulose from plants and algae is not pure and contains impurities like unwanted toxic heavy metals, glucan, endotoxins, lignin, alkaloids, hemicelluloses, and pectin. Therefore, the plant and algal-derived nanocellulose require purification through chemical and physical approaches prior to their use for different applications (Sano et al., 2010; Picheth et al., 2017). The common methods used to extract nanocellulose from a cellulosic material contain several problems, such as the higher amount of acid wastewater generation for the acid hydrolysis, high energy utilization for the mechanical process, and elevated reaction duration for enzymatic hydrolysis (Phanthong et al., 2018; Skiba et al., 2020). As compared to the large-scale production of CNF and CNC, the BNC production is only limited to laboratory-scale due to the high cost of production medium and growth maintenance of BNC-producing microbial strains as well as the low yield (Ul-Islam et al., 2020). Regardless of the development of various bioreactors for BNC production, the highest productivity with aerosol bioreactor is only 0.38 g/(L/) (Lee et al., 2014). Although pristine nanocellulose is non-toxic and non-genotoxic, the chemically modified nanocellulose might cause complications when used for biomedical applications (Chinga-Carrasco, 2018). For example, an earlier study reported that the addition of the dialdehyde group to nanocellulose enhanced the gene expression of tumor necrosis factor (TNF-) and induced inflammation at the target site (Kollar et al., 2011). Moreover, the bacteria-derived nanocellulose might contain contamination of unwanted lipopolysaccharides, which may cause inflammation at the target site (Chinga-Carrasco, 2018).

### *In vivo* Biodegradation of Nanocellulose

Cellulose has excellent mechanical and biocompatible properties; however, it is not degradable or degrades very slowly in animals due to a lack of cellulase enzyme (Lin and Dufresne, 2014); nevertheless, slow degradation of nanocellulose could be advantageous by favoring a continuous drug release, which could be further optimized (Patel et al., 2019). Furthermore, the innate features of nanocellulose such as hydration, swelling, and crystallinity may affect not only the degree of degradation but also the absorption and immune response (Lin and Dufresne, 2014). For example, in canine, cellulose, and cellulose derivatives, the rate of degradation depends mainly on the cellulose chemical derivation and crystalline form. The oxidized cellulose is more vulnerable to hydrolysis and, therefore, could be degraded by the human body (Luo et al., 2013). Mineral acids, such as phosphoric acid, sulfuric acid, and hydrochloric acid, hydrolyze the nanocellulose crystals (Endes et al., 2016). The composites of nanocellulose with metal oxide (Fe_2_O_3_, graphene oxide, ZnO, and TiO_2_) have been utilized for improving the degradation rate (Shak et al., 2018). The degradation of nanocellulose could be enhanced by introducing *N*-acetylglucosamine residues and by incorporating cellulase enzymes, making it applicable for several medical purposes (Bacakova et al., 2019).

## Conclusion and Prospects of Using Nanocellulose in Treating Bone-Related Diseases

Over the past couple of decades, extensive efforts have been devoted to nanocellulose research. The nanocellulose research is primarily focused on exploring low-cost substrates, developing advanced and facile preparation strategies, and exploring new application areas. To date, different forms of nanocellulose, especially BNC, have been extensively explored for various biomedical applications, especially for developing tissue engineering scaffolds. Nanocellulose is not only suitable for the development of soft tissue scaffolds such as skin, neural, and other tissues, it can be effectively utilized for the development of hard tissues such as bone and cartilage when combined with other materials to impart it compatible compression and shape.

Advanced therapeutic strategies for treating bone diseases could be developed by using nanocellulose both as the scaffold and a matrix or carrier. Due to its high mechanical strength and biocompatible nature, nanocellulose could be used as a suitable biomaterial for scaffolding and bone regeneration and development. The deposition of metallic nanoparticles on nanocellulose would further impart antimicrobial activity to the scaffold. Nanocellulose could also be used as an immobilizer for macromolecules, such as DNA, protein, and enzymes. It can also be utilized to enhance the production of secondary metabolites in highly medicinal plants by activating the key genes. The loading of luminescent nanomaterial on nanocellulose could be a novel approach for its use as a fluorescent probe and for photoelectric and photothermal applications. Similarly, nanocellulose could serve as a matrix or carrier for the immobilization of drugs and nanoparticles for avoiding the immune response and for the controlled release of advanced therapeutic molecules and nanomaterials. For targeted delivery of therapeutic molecules/metallic components to treat bone diseases, there must be an appropriate nanocarrier that could be used to encapsulate the therapeutic entities in order to avoid the immune response and early elimination from the body. Certain nanocarriers that protect nanodrugs and deliver to a specific target site encounter several limitations; thus, an alternative approach is required. Nanocellulose could be a suitable nanocarrier for metallic nanoparticles to treat bone diseases. For example, nanocellulose biocompatible and works well as the stabilizing agent for metallic nanoparticles by preventing their agglomeration and maintaining their morphological stability. Nanocellulose can be implanted over metallic nanodrugs through chemical reduction or physical adsorption (electrostatic association). Furthermore, the attachment of osteoblastic antigen on the surface of nanocellulose can enhance the potency of targeted delivery of metallic nanodrugs and can become a novel approach to treat damaged bone or bone-related diseases. Similarly, a controlled drug release is an important phenomenon that gains high importance in cases where the timely release of drug is required. For instance, an anticancer drug bendamustine requires controlled release after oral delivery; therefore, nanocellulose can be helpful to regulate the drug release inside the body. Although nanocellulose is largely considered to be used for conventional FDA approved drugs, it can also be used for the delivery of more advanced therapeutic entities such as siRNAs. The siRNAs-based therapies are considered very effective; however, their poor stability in the biological environment is a serious concern. Therefore, nanocellulose-based materials can be used to provide protection from degradation and enhancing cellular uptake.

Although the above discussion justifies the importance and potential use of nanocellulose in treating bone-related diseases, further investigation on cell-scaffold interaction, *in vivo* degradation, and resorption of degradation products is required. Moreover, immunological analysis of nanocellulose-based scaffolds is required for a complete understanding of their effects on both innate and adaptive immunity. To this end, a detailed investigation on the identification of molecular mechanisms present in the immune system for recognition of nanocellulose and the activation of associated downstream signaling pathways will pave the way to rationally develop biologically safe nanocellulose-based scaffolds for clinical applications.

## Author Contributions

SK and RS wrote the original draft. SK, DH, MAS, GN, QB, SM, and MWU revised and modified the manuscript. MX, MWU, and HB supervised and edited the manuscript. All authors contributed to the article and approved the submitted version.

## Conflict of Interest

The authors declare that the research was conducted in the absence of any commercial or financial relationships that could be construed as a potential conflict of interest.
